# Elucidating sensing mechanisms of a pyrene excimer-based calix[4]arene for ratiometric detection of Hg(ii) and Ag(i) and chemosensor behaviour as INHIBITION or IMPLICATION logic gates†

**DOI:** 10.1039/d0ra04092d

**Published:** 2020-06-08

**Authors:** Julio Rodríguez-Lavado, Alejandro Lorente, Erick Flores, Andrés Ochoa, Fernando Godoy, Pablo Jaque, Claudio Saitz

**Affiliations:** Departamento de Química Orgánica y Fisicoquímica, Facultad de Ciencias Químicas y Farmacéuticas, Universidad de Chile Olivos 1007 Independencia Santiago Chile julio.rodriguez@ciq.uchile.cl; Departamento de Química de Los Materiales, Universidad de Santiago de Chile Libertador Bernardo ÓHiggins 3363 Santiago RM Chile

## Abstract

This article reports the synthesis and characterisation of two lower rim calix[4]arene derivatives with thiourea as spacer and pyrene or methylene–pyrene as fluorophore. Both derivatives exhibit a fluorimetric response towards Hg^2+^, Ag^+^ and Cu^2+^. Only methylene–pyrenyl derivative 2 allows for selective detection of Hg^2+^ and Ag^+^ by enhancement or decrease of excimer emission, respectively. The limits of detection of 2 are 8.11 nM (Hg^2+^) and 2.09 nM (Ag^+^). DFT and TD-DFT computational studies were carried out and used to identify possible binding modes that explain the observed response during fluorescence titrations. Calculations revealed the presence of different binding sites depending on the conformation of 2, which suggest a reasonable explanation for non-linear changes in fluorescence depending on the physical nature of the interaction between metal centre and conformer. INHIBITION and IMPLICATION logic gates have also been generated monitoring signal outputs at pyrene monomer (395 nm) and excimer (472 nm) emission, respectively. Thus 2 is a potential primary sensor towards Ag^+^ and Hg^2+^ able to configure two different logic gate operations.

## Abstract 2

This article reports the synthesis and characterisation of two lower rim calix[4]arene derivatives with thiourea as spacer and pyrene or methylene-pyrene as fluorophore.

## Graphical abstract

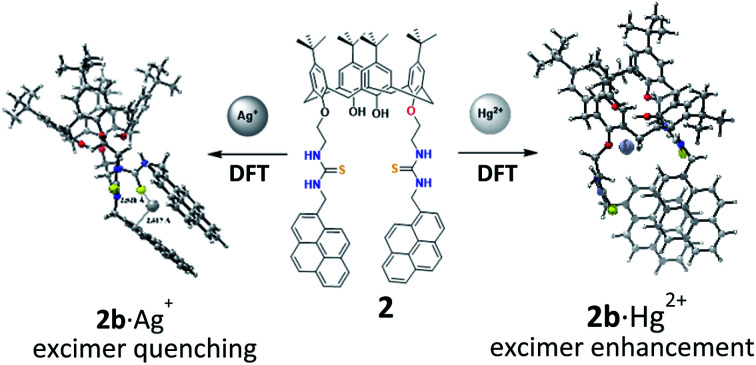

## Introduction

Transition metal ions play an important role in a wide range of chemical reactions, including biological metabolism and many other natural or anthropogenic processes.^1–4^ Hence, much effort has been made in order to develop chemosensors capable of their qualitative or quantitative determination.^1,5–7^ Silver ions are widely studied because of their antimicrobial activities,^8^ applications in photography, electronics and medicinal chemistry (*e.g.* silver nanoparticles).^9^ However, silver contamination can occur through a variety of sources including volcanoes, gold mining or fossil fuel combustion.^10^ As a result of that, excess of silver ions can lead to bioaccumulation and intoxication,^11^ resulting in adverse effects affecting kidney function, immune and cardiovascular systems, and inactivation of sulfhydryl enzymes.^12^ Additionally, mercury is one of the most toxic and environmentally widespread contaminants, causing serious damage to living organisms and the environment. Consequently, when absorbed in tissues it can result in damage to the central nervous system and liver, leading to motor and cognitive disorders.^13,14^ Within the different toxic species of mercury, its divalent form can lead to severe brain and kidney damage, even though it cannot cross the blood brain barrier.^15^ Consequently, the development of new methods providing high sensitivity and selectivity to detect Ag^+^ or Hg^2+^ are highly welcomed in many fields.

In this context, different techniques for detection of such contaminants have already been reported.^3,16–25^ Among them, fluorescence-based sensors are particularly interesting over other techniques due to their simplicity for implementation, fast response, local observation, selectivity and sensitivity.^1,5^

In particular, fluorescent ratiometric detection has become one of the most powerful tools for molecular sensing. Since it allows measurement of emission intensities at two different wavelengths, so that the ratios of signals will be independent of the environmental effects. Ratiometric-based chemosensors rely on the principle that it improves the dynamic response according to the change of intensity ratios, thus establishing the internal standard. Compared with conventional chemical analysis, the ratiometric method has thus a higher selectivity and more simplicity in the detection of ligand–receptor binding by observing merely the enhanced fluorescence of the acceptor.^26–29^

An approach generally used for this kind of molecular sensors consist of an ion recognition moiety, required for the selective binding to the substrate, coupled to fluorophore unit, providing a moniterable signal. Both units are typically linked to a molecular scaffold.^30^

In order to develop recognition moieties, calix[4]arenes have been widely used as molecular scaffold due to their ease and controlled functionalisation.^5,31^ Furthermore, they provide a suitable platform to develop a cavity in which selectivity can thus be finely tuned.^32^ For example, by the use of triazoles, ureas, amides or thioureas as linker between signalling moiety and the molecular scaffold. The latter has shown to be a highly versatile recognition moiety for metal cations,^33^ as well as organic anions^34^ and DNA,^35–37^ due to the variety of possibilities to bind the substrate, *i.e.* the presence of nitrogen and sulphur coordinating atoms are able to bind cations by either *through-bond* or *through-space* interactions, while NH hydrogen bond donor groups are able to stabilize anions and DNA by H-bonding interactions.^33,38–40^

Among the wide range of fluorescent moieties reported, pyrene has attained much attention due to its well-known photophysical properties such as high quantum yields, well-defined absorption and emission bands, long lifetime and the possibility to form excimers.^41,42^ These unique properties have inspired the development of pyrene-based sensors due to providing several sensing modes, by monitoring either the monomer (*ca.* 370–390 nm) or excimer (*ca.* 470–490 nm) emission bands, or the ratio of both. The ratio of the intensities of excimer and monomer emission is very useful for analyte sensing because it is highly sensitive to the conformational changes of the pyrene-anchored to any molecular architecture, which are induced by the interaction mode between the analyte and the recognition moiety.

Nevertheless, many studies that have used aforementioned supramolecular approach for the development of chemosensors do not explore in detail the different binding modes available within the cavity. Although many succeed in the determination of the host–guest stoichiometry,^43,44^ or are able to provide crystal structures,^22,45^ a significant part of them only propose a most plausible binding mode. Furthermore, whether different equilibriums are reached upon increasing concentration of guest, then the determination of the binding constant turns challenging. In this context, quantum chemical methods are an alternative tool to investigate the electronic and molecular structures of host and respective host–guest complexes, unveiling the physical nature of the interactions and their impact on the fluorescence response, giving a different standpoint that goes beyond the typically proposed mechanisms. Potentially, this type of studies could allow to gain a new insights into the calix[4]arenes-based chemosensors. Many quantum-chemical-based studies have focused their attention on the formation of isolated pyrene–excimer^46^ and their properties as well as in the investigation on the binding of different analytes using calix[4]arene scaffolds.^47,48^ More recently, density functional theory (DFT) has been employed to understand the sensing mechanism of lysine through the formation of the intermolecular pyrene–excimer.^49^

Herein we present the synthesis and characterisation of two calix[4]arene derivatives composed by pyrene units connected through a thiourea-based spacer. We studied their photophysical properties, their ability to show excimer emission, and their fluorescent response to different cations. Furthermore, we carry out a quantum chemical study based on density functional theory (DFT) and time-dependent (TD-DFT) frames, aimed to explain the experimental observations through the identification of the specific interactions that lead to the stabilization of different ligand conformers and their respective metal complexes. Additionally, operation of one of the sensors as INHIBITION or IMPLICATION logic gate is also described,^50^ since it gives different fluorescent outputs when it is treated with Ag^+^ and Hg^2+^.

## Results and discussion

### Synthesis

**Scheme 1:**
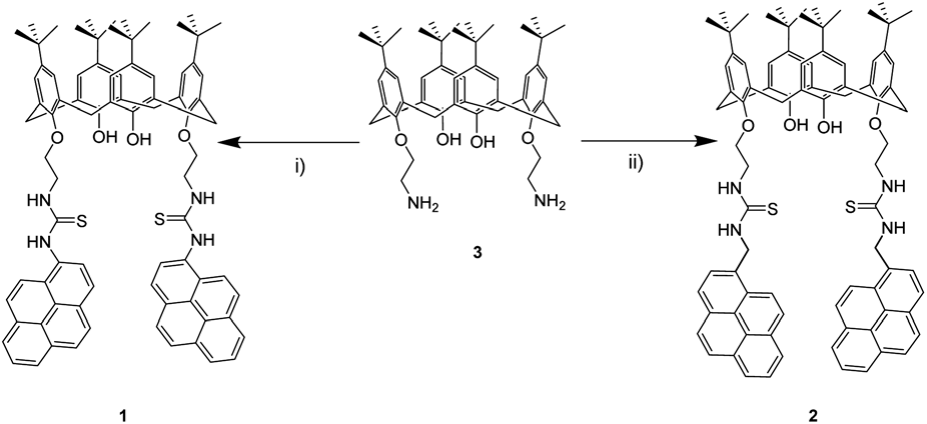
Reagents and conditions: (i) 1-pyreneisothiocyanate, Et_3_N, dry CH_2_Cl_2_, rt, 48 h, (39%), (ii) 1-(isothiocyanatomethyl)pyrene, Et_3_N, dry CH_2_Cl_2_, rt, 48 h (33%).

The synthetic route designed for chemosensors 1 and 2 takes advantage of the thiourea forming reaction within amines and isothiocyanates. Pyreneisothiocyanates and calix[4]arene 3 derivatives were prepared by reported methodologies.^51–53^ The reactions were carried out in dry CH_2_Cl_2_/triethylamine solution, and stirred at room temperature for 48 hours (Scheme 1). An advantage of this synthetic strategy is that the final products precipitated. By simply filtering and washing off these precipitates, products 1 and 2 were isolated in moderate yields without further purification. It is noteworthy to mention that 1 synthetic route was firstly attempted by coupling 1-aminopyrene derivative to the analogue isothiocyanate derivative from 3. Nevertheless, this route did not yield the desired products, most likely due to the low basicity of the aromatic amine.

^1^H and ^13^C NMR spectra were consistent with the proposed structures. In both cases, two differentiated aromatic signals related to pyrene (8.3–7.4 ppm) and calix[4]arene units (6.7–6.4 ppm) are observed. In our case, a characteristic pair of doublets at 3.3 and 2.4 ppm for compound 1 and 3.3 and 2.7 ppm for 2, whose coupling constants are around 13.0 Hz, support the *cone* conformation of 1 and 2. For compound 2, a singlet at 5.3 ppm is assigned to the methylene bridge between the thiourea and pyrene units. Besides, in ^13^C NMR spectra, a signal observed at around 182 ppm for both molecules confirmed thiocarbonyl bond formation. Additionally, high-resolution mass spectrometry is in agreement with the proposed molecular structures (see ESI†).

### Spectroscopic properties

**Fig. 1:**
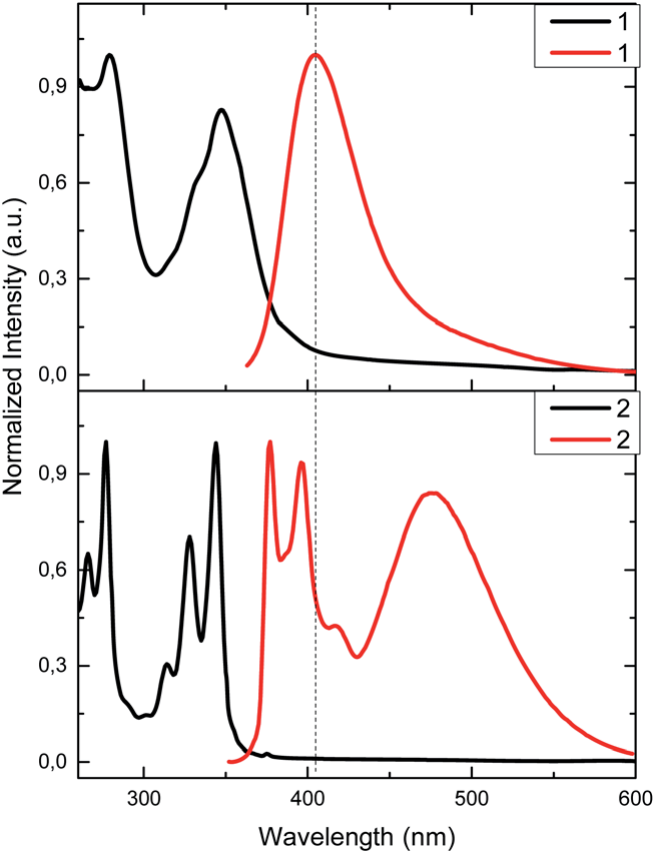
Normalized absorption (9.0 μM, black) and emission (1.15 μM, *λ*_exc_ = 340 nm, red) in CH_3_CN/DMSO (99 : 1) solutions for 1 and 2.

Photophysical properties of 1 and 2 were studied by UV-vis and fluorescence spectroscopy in acetonitrile/DMSO (99 : 1) solutions (Fig. 1). Calix[4]arene 1 exhibits a well-defined absorption band at 280 nm and two overlapped bands at 332 and 347 nm instead of pyrene fine structure. In 2, sharper bands are observed at 265, 276, 314, 327 and 344 nm. Bands in the range from 265 up to 280 nm are most likely related to calix[4]arene derivatives, while the bands in the range from 314 up to 347 nm are related to pyrene moieties, retaining its typical absorption fine structure.^54^

Although there is a slight difference between chemical structures of 1 and 2, fluorescence spectra show very different emission profiles when excited at 340 nm. In the case of 1, the characteristic emission spectrum of pyrene monomer is replaced by a featureless profile; a broad band with a maximum at 406 nm is observed. This emission is most likely related to the pyrene-thiourea unit, as it can be seen in previous reports that study analogous fluorophore structures, plausibly due to some rotational degree of freedom.^18,55,56^ This similarity in emission profiles suggests that calix[4]arene core has negligible effect in the emission properties of pyrene. On the other hand, 2 presents three main emission bands. Two of them appear at 377 and 395 nm, and are related to the pyrene emission as noted earlier in its emission spectrum.^54^ The third one, at 472 nm, can be attributed to the relaxation of a pyrene–excimer formed, most likely due to CH_2_ bridge. This type connectivity brings an additional degree of freedom to pyrene, favouring the formation of excimers as seen in previous reports.^19,57–60^

In order to demonstrate that pyrene units are forming an intramolecular excimer rather than an intermolecular one, we performed dilution studies for 2 in the concentration range from 1 × 10^−5^ to 1 × 10^−7^ M (Fig. S1†). Fluorescence intensity ratio of excimer/pyrene (*I*_472_/*I*_395_) keeps constant at 0.90 suggesting that the excimer emission observed arises from an intramolecular interaction of pyrenes.

### Screening with metal ions and ratiometric detection

Compounds 1 and 2 incorporate heteroatoms able to act as polydentate ligands (O, N and S), which may interact differently with metal ions. The binding properties of 1 and 2 were investigated by fluorescence spectroscopy in CH_3_CN/DMSO (99 : 1). The studied cations include alkaline, alkaline-earth and transition metals (Li^+^, Na^+^, K^+^, Mg^2+^, Ca^2+^,Cd^2+^, Co^2+^, Cu^2+^, Mn^2+^, Ni^2+^, Zn^2+^, Fe^2+^, Ag^+^, Hg^2+^ and Pb^2+^) as perchlorate salts. The screening experiments were carried out by adding 2 equivalents of each cation.

**Fig. 2:**
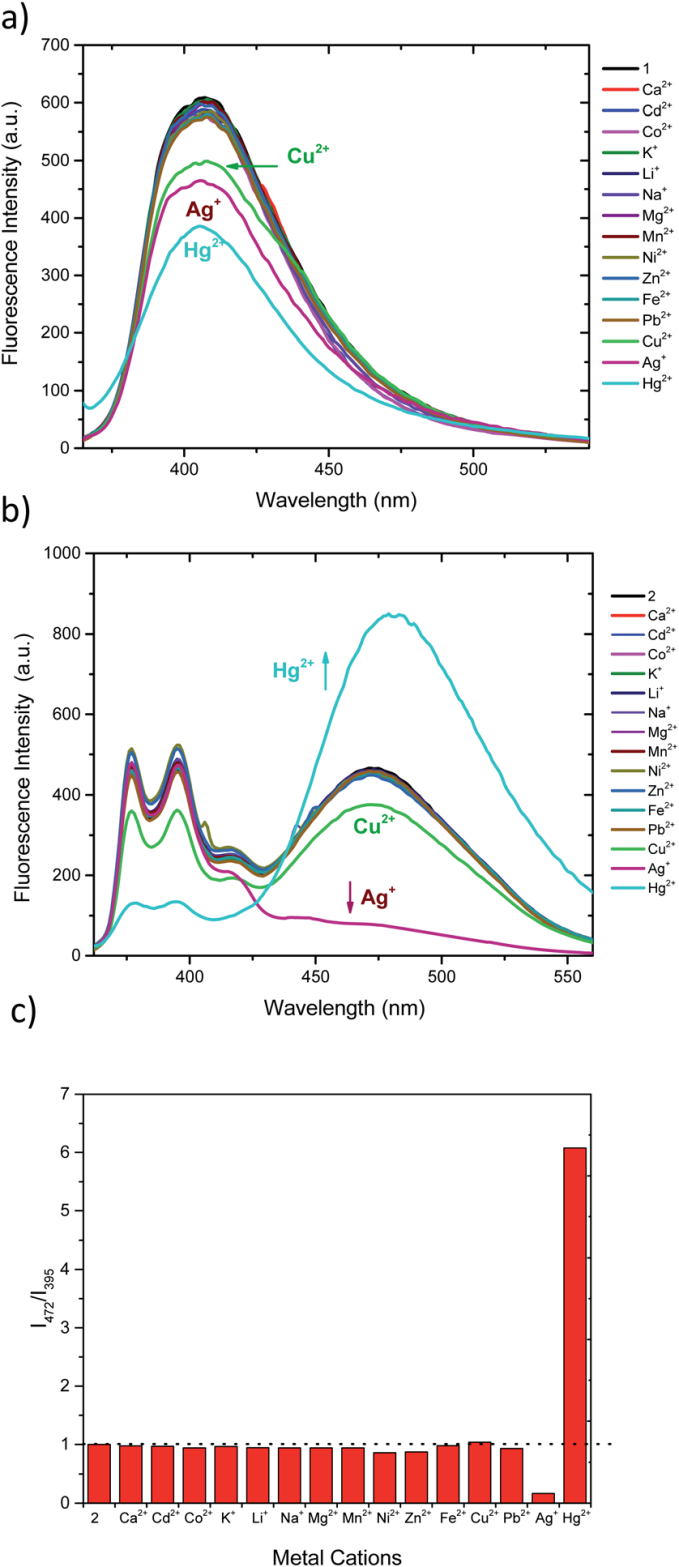
Fluorescence response of 1 (a) and 2 (b) (1.15 μM in CH_3_CN/DMSO 99 : 1) upon addition of 2 eq. of studied cations. (c) Ratiometric change of chemosensor 2 (*I*_472_/*I*_395_) upon addition of studied cations.

Both sensors display fluorescence changes upon addition of Ag^+^, Hg^2+^ and Cu^2+^ (Fig. 2a and b). In the case of 1, deactivation of the fluorescence at 406 nm is observed for the aforementioned cations.^61,62^ However, the observed response does not allow for ratiometric detection, since no new band is observed. Furthermore, the similar response to the three cations may limit the applicability of 1 as selective chemosensor. Conversely, chemosensor 2 shows a completely different response. Upon addition of Ag^+^ the emission intensity of pyrene excimer band (472 nm) decreases (Fig. 2b), while addition of Hg^2+^ results in an increase of intensity of excimer emission and a decrease in the monomer emission. When treated with Cu^2+^ a slight decrease of intensity of all bands is perceived. In order to see whether higher selectivity of 2 within these cations can be obtained, ratiometric variation (*I*_472_/*I*_395_) of fluorescence intensity of 2 with the studied cations is represented in Fig. 2c. Interestingly, under this analysis it can be seen that Cu^2+^ no longer is a significant cation to be detected by 2, showing only a slight perturbation (<5%) similar to that of Pb^2+^, Ni^2+^ or Zn^2+^.^27,28^ Interestingly, probe 2 shows no significative interference when a mixture of all the cations at the same concentration are present (Fig. S7a†) and, the interference studies using Hg^2+^ and Ag^+^ interacting with each other, show that the Hg^2+^ output is predominant (Fig. S7b†), which is in agreement with the observed Log *K*_app_.

### Titrations, limits of detection and *K*_app_

**Fig. 3:**
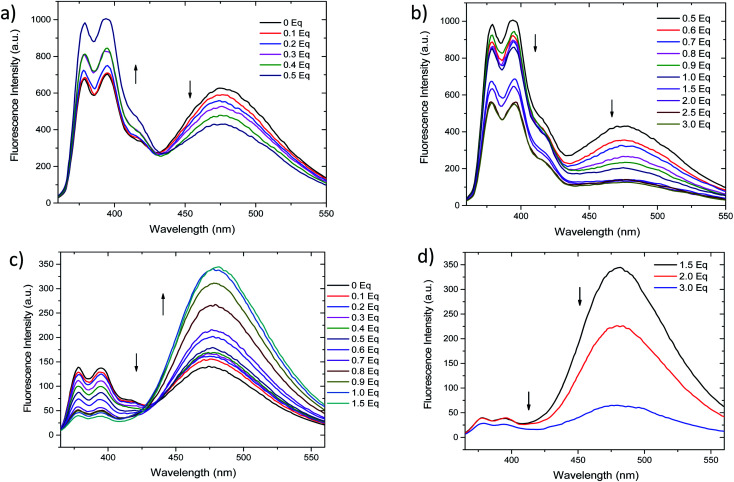
Titration curves for 2 (1.15 μM in CH_3_CN/DMSO 99 : 1) with Ag^+^ and Hg^2+^. (a) Ratiometric change of fluorescent signals in the range from 0–0.5 eq. of Ag^+^. (b) Fluorescence quenching in the range from 0.6–3.0 eq. of Ag^+^. (c) Ratiometric change of fluorescent signals in the range from 0–1.5 eq. of Hg^2+^. (d) Fluorescence quenching in the range of 1.5–3.0 eq. of Hg^2+^.

Fluorescent titrations of the compound 2 with Ag^+^ and Hg^2+^ (0 to 3 equivalents) were performed. As depicted in Fig. 3a, addition of 0.1 to 0.5 equivalents of Ag^+^ results in ratiometric variation of monomer and excimer bands, in which monomer fluorescence (377 and 395 nm) increases while excimer band (472 nm) decreases. Further increase in Ag^+^ concentration (0.6 to 3.0 equivalents, Fig. 3b) results in a different variation of fluorescence, since all bands simultaneously decrease in intensity. On the contrary, although the addition from 0.1 to 1.5 equivalents of Hg^2+^ the fluorescence intensities also change ratiometrically (Fig. 3c), in this case monomer fluorescence is quenched, while pyrene–excimer fluorescence is enhanced. These differentiated changes allow 2 to discriminate between Ag^+^ and Hg^2+^ species. Upon further addition of Hg^2+^ (1.5 to 3.0 equivalents), similarly as with Ag^+^, intensity of all emission bands decreases (Fig. 3d). Additionally, we also confirmed that the perchlorate anion had no effects in the fluorescence (Fig. S2†). In order to get better insight of the fluorescence response as cation concentration increase ^1^H NMR titrations were attempted. However, they result unsuccessful due to the low solubility of 2 in DMSO-*d*^6^, CDCl_3_ and CD_3_CN. In the following section this issue will be address by using computational approaches.

The limits of detection (LODs) of 2 were estimated based on the fluorescence titration data (Table 1 and Fig. S3†). LODs were found to be 8.11 nM for Hg^2+^ and 2.09 nM for Ag^+^.^63^ These values result almost 10 fold lower than similar reported calix[4]arene based systems^64–67^ or other fluorescent chemosensors.^68,69^ Comparative data are displayed in Table S1.†

**Table 1:** Log *K*_app_, Hill Coefficient, and LOD of studied metal complexes

Complex	Log *K*_app_	*n*	LOD (nM)
2·Ag^+^	9.64 ± 0.01	1.56 ± 0.02	2.09 ± 0.44
2·Hg^2+^	10.64 ± 0.15	1.71 ± 0.01	8.11 ± 0.58

Titration data were also used to determine association constants of 2 with Hg^2+^ and Ag^+^. Among the different methods normally applied to calculate them, Benessi–Hildebrand equation cannot be applicable in this case due to the observation of two different fluorescent-based sensing mechanisms.^70^ Non-linear fitting was also evaluated for 2 titration curves with Ag^+^ and Hg^2+^, resulting in high deviations for similar reasons.^70^

Additionally, we attempted to clarify complexation stoichiometry by using the method of continuous variation (Job's Plot, Fig. S4 and S5†), even though we are conscious of its limitation in many supramolecular systems.^71,72^ Unfortunately, maxima positions do not correlate with results showed by similar previously described calix[4]arene-based chemosensors, in which only a single binding site and/or conformation have been preferred.^63^

For these reasons, apparent association constants *K*_app_ for 2·Hg^2+^ and 2·Ag^+^ were calculated by linear fitting according to the Hill equation, being Log *K*_app_ 10.64 and 9.64, respectively (Table 1 and Fig. S6†).^73,74^ The data suggest that 2 interact stronger with Hg^2+^ than with Ag^+^. This method has been used on other pyrene-based sensors involving two succinic imide labelled pyrenes that coordinate with Hg^2+^. This system, with a 2 : 1 stoichiometry, is similar to ours since calix[4]arene is assembling two thiourea-pyrene units.^73^ The obtained Hill coefficients are *n* = 1.7 and 1.5 when adding Ag^+^ and Hg^2+^, respectively.^75,76^

### Quantum chemical calculations

We carried out a computational study based on DFT and TD-DFT calculations aimed to find 2 conformers and their respective Ag^+^ and Hg^2+^ complexes, together with the identification of stabilizing interactions and spectral features in order to explain the experimental sensing behaviour and propose a sensing mechanism.

#### Molecular geometries: 2 and 2·Ag^+^/2·Hg^2+^ complexes

**Fig. 4:**
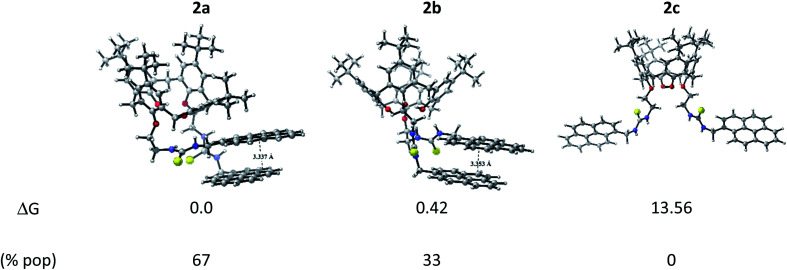
Optimized conformers of 2 and their relative Gibbs free energy (in kcal mol^−1^) and Boltzmann population in acetonitrile.

Three conformers were found for calix[4]arene derivative 2 in gas-phase as displayed in Fig. 4. Two of them (labelled as 2a and 2b in Fig. 4) are the most stable as is unveiled by the relative Gibbs free energy values in solution (which is given by the sum of the electronic, thermal and entropic contributions and solvation energies and it is referred to the most stable conformation). Notice that both conformers present pyrene monomers nicely oriented to formation of parallel-displaced ground state pyrene dimer, with inter-plane distances of 3.337 and 3.353 Å for 2a and 2b, respectively, which is slightly shorter than previous reports for isolated pyrene dimer (*e.g.* 3.45 Å).^46,77^ This type of orientation has been shown to play a key role in the excimer formation. Consequently, it can provide a contribution to the appearance of the broad and structureless excimer fluorescence band, which is red-shifted with respect to monomer fluorescence. Moreover, we included the relative population (% pop) in solution following the standard Boltzmann distribution analysis. While 2a presents a 67%, 2b a 33%, therefore, 2 exist in solution as mixtures of 2a and 2b in an equilibrium ratio of 2 : 1. This result opens the possibility to explore different binding mode towards metallic cations, which can exhibit varied fluorescence response in the development of 2-based chemosensor. 2c conformation showed to be an unstable one, concomitantly, the only option to excimer formation is by an intermolecular pathway due to that the pyrene units are away from each other. Three conformations were also found for calix[4]arene derivative 1 (see Fig. S14†), but contrary to 2, it exists in solution in its most stable conformation 1a as predicted by Boltzmann distribution analysis. Notice that 1a presents a tilted T-shaped configuration among the pyrene moieties in its ground state, which contrasts with a parallel-displaced conformation that stabilizes the ground state of 2a and 2b. This feature explains the appearance of the excimer emission band in 2. Additionally, the basis set superposition error (BSSE)-corrected instantaneous interaction energies values, Δ*E*_int_(BSSE), for this fragments are higher in 2a (−12.55 kcal mol^−1^) and 2b (−12.70 kcal mol^−1^) than 1a (−8.45 kcal mol^−1^) by around 4.1 kcal mol^−1^, unveiling that the structures 2a and 2b are more rigid than 1a, this fact explains the retention of fine structure in its absorption/emission spectra of derivative 2.

**Fig. 5:**
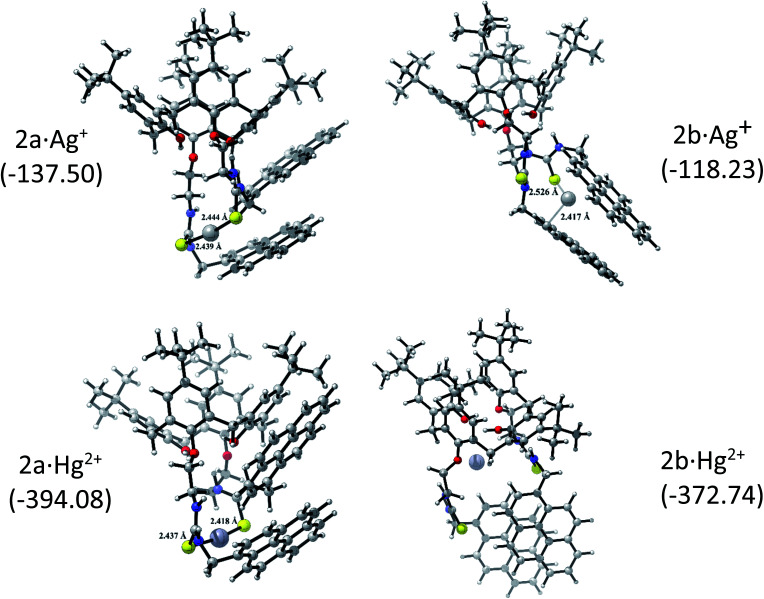
Optimized 2a/2b·Ag^+^ and 2a/2b·Hg^2+^ complexes and the BSSE-corrected interaction energy, Δ*E*_int_(BSSE) (the values are given in parenthesis in kcal mol^−1^).

The corresponding 2·Ag^+^ and 2·Hg^2+^ complexes with each 2a and 2b conformations were also fully optimized (as displayed Fig. 5) and confirmed as local minima. Δ*E*_int_(BSSE) data are also included. The procedure followed here was to start with the same initial geometry, which consists in locating each cation inside the cavity of conformer 2a and 2b, until the fully relaxed structure is reached for each complex. It can be noted that the binding mode with 2a is quite similar for each metal centre. Contrarily, the binding mode with 2b is totally different. In accord with Δ*E*_int_(BSSE) values, it is found that Hg^2+^ binds to 2a stronger than Ag^+^, being in agreement with experimental *K*_app_. Both 2a·Ag^+^ and 2a·Hg^2+^ complexes are mainly characterised by an interaction between S atoms and metal centre. The bond lengths are close to the sum of the corresponding covalent radii (2.500 Å and 2.370 Å for 2a·Ag^+^ and 2a·Hg^2+^, respectively). This feature is an indicative that *through-bond* interactions or covalent-type (delocalization) in conjunction with the dispersion ones prevail over other interactions in the stability of 2a·Ag^+^/Hg^2+^ complexes. Again, Hg^2+^ binds to 2b stronger than Ag^+^. Nevertheless, the structures showed noticeable differences. While 2b·Ag^+^ is cooperatively stabilized by Ag–S *through-bond* and cation⋯π interactions in conjunction with dispersion interactions, 2b·Hg^2+^ is mainly stabilized by *through-space* interactions (localization) together with dispersions interactions. Since N and O atoms enclose Hg^2+^ cation inside the cavity of 2b, the distances are in the range of 3.048 to 3.480 Å, which are longer than the sum of their van der Waals radii (3.10 Å and 3.07 Å for Hg⋯N and Hg⋯O, respectively). This feature together with the high interaction energy suggests an electrostatic nature as stabilizing force in 2b·Hg^2+^ complex. Overall, both metal cations interact more strongly with 2a than 2b. It must be emphasized that the pyrene units are less stacked in the metal complexes than in 2a and 2b, except in 2b·Hg^2+^, where the stacking is considerably more accentuated and plausibly more rigid due to the *through-space* interactions that stabilizing it.

#### Spectral features

We only computed the vertical excitation property due to that is less computationally demanding than to carry out excited state optimisations. The vertical electronic excitation energies together with absorption properties, such as oscillator strength (*f*) and the main molecular orbitals contribution involved in the electronic transitions were computed within the TD-DFT/SMD framework for 2a, 2b and the respective metal complexes. The corresponding data are quoted in Table 2. Firstly, the computed transition energies agree with the experimental absorption energy of pyrene dimer (3.70 eV (ref. 78 and 79)). Our attention has been focused on this absorption band, which is associated with the electronic transition that originates the fluorescence response employed to ratiometric fluorescent detection of Ag^+^ and Hg^2+^. In most of the cases, we can observe that the electronic transition involves the two highest occupied molecular orbitals (HOMO−1, H−1, and HOMO, H) and the two lowest unoccupied molecular orbitals (LUMO, L, and LUMO+1, L+1). As can be seen in Fig. S15,† both occupied and unoccupied molecular orbitals show certain bonding character in 2a, which is opposite to the character found in the isolated pyrene dimer: H is antibonding while L is bonding. This feature is plausibly due to the shorter inter-plane distance in 2a than in free pyrene dimer, which, on the other hand, is favoured by the use of calix[4]arene as molecular scaffold. An interesting spectral property that provides understanding on the absorption/emission probability is the oscillator strength. Calculated *f* showed to be relatively high, it can be noted that the presence of Ag^+^ in the complexes increases *f* while Hg^2+^ decreases the values of *f* with respect to the conformer 2a and 2b. 2b·Hg^2+^ deserves special attention because TD-DFT calculations unveil an electronic transition at 465 nm (2.67 eV) with *f* of 0.0480. Its character is given from inner together with H molecular orbital to L and L+1. This feature is very significant due to that the pyrene excimer fluorescence is observed at this region. This fact could explain an enhancement of the fluorescent response in the first ratiometric fluorescent probe for Hg^2+^ at low concentration regime showed in Fig. 3c.

**Table 2:** Electronic transition energy (*E*), wavelength (*λ*), oscillator strength (*f*) and main contributions

	*E* (eV)	*λ* (nm)	*f*	Contributions
2a	3.77	329	0.5068	H → L+1 (56%)
				H−1 → L (44%)
2a·Ag^+^	3.76	330	0.5633	H−1 → L (59%)
				H−1 → L+1 (23%)
2a·Hg^2+^	3.74	332	0.4972	H → L+2 (68%)
				H → L (17%)
2b	3.84	323	0.7064	H−1 → L+1 (38%)
				H → L (29%)
				H → L+1 (17%)
2b·Ag^+^	3.77	329	0.8054	H → L+1 (58%)
				H−1 → L (39%)
2b·Hg^2+^	3.42	363	0.6349	H → L+1 (81%)
				H−1 → L+1 (12%)

To the light of our results, we propose that the first sensing mechanism for both cations could be originated by the formation of the complex with 2b conformer of 2. In the case of silver, upon interaction of 2b with Ag^+^, the π–π stacking pyrene rings breaks and results in excimer quenching and monomer enhancing. While a rigid pyrene-excimer is formed 2b·Hg^2+^ due to the presence of the *through-space* interactions, results in an enhancement of the excimer fluorescence (by localizing charge into the excited state) together with quenching of monomer emission. In the second sensing mechanism is mainly explained with the presence of metal complexes with 2a. We propose that the stabilizing *through-bond* interactions in both complexes are responsible to quench both the monomer and excimer fluorescence, by delocalizing charge into the corresponding excited states.

DFT/TD-DFT predictions suggest future photophysical and time dependent fluorescent investigations on 2b-based chemosensor in all concentration regimes to confirm the sensing mechanism proposed here.

### Application of 2 as molecular logic gate

Fluorescent chemosensors that are able to perform logic operations have attracted much attention due to its potential applications in biosensing and diagnosis, contaminant determination or molecular computation.^80–82^ In general, their application as molecular logic gates is possible when two or more inputs give different fluorescent outputs or sensing mechanism as unveiled our results. Among the sixteen Boolean operations that can be configured, six of them are rather trivial in terms of fluorescent output or molecular design.^83^ Within the rest, most of reported molecular logic gates are able to perform AND, OR, XOR operations.^50,84,85^

In the case of INHIBITION gates, the “1” output is generated when only one input is present without the other input, *i.e.* one input has the power to activate the whole system.^84^ Nevertheless, molecular design of IMPLICATION gates is more challenging since fluorescence output has to be “1” in the absence of both inputs and only be quenched by one of the inputs.^83^

**Fig. 6:**
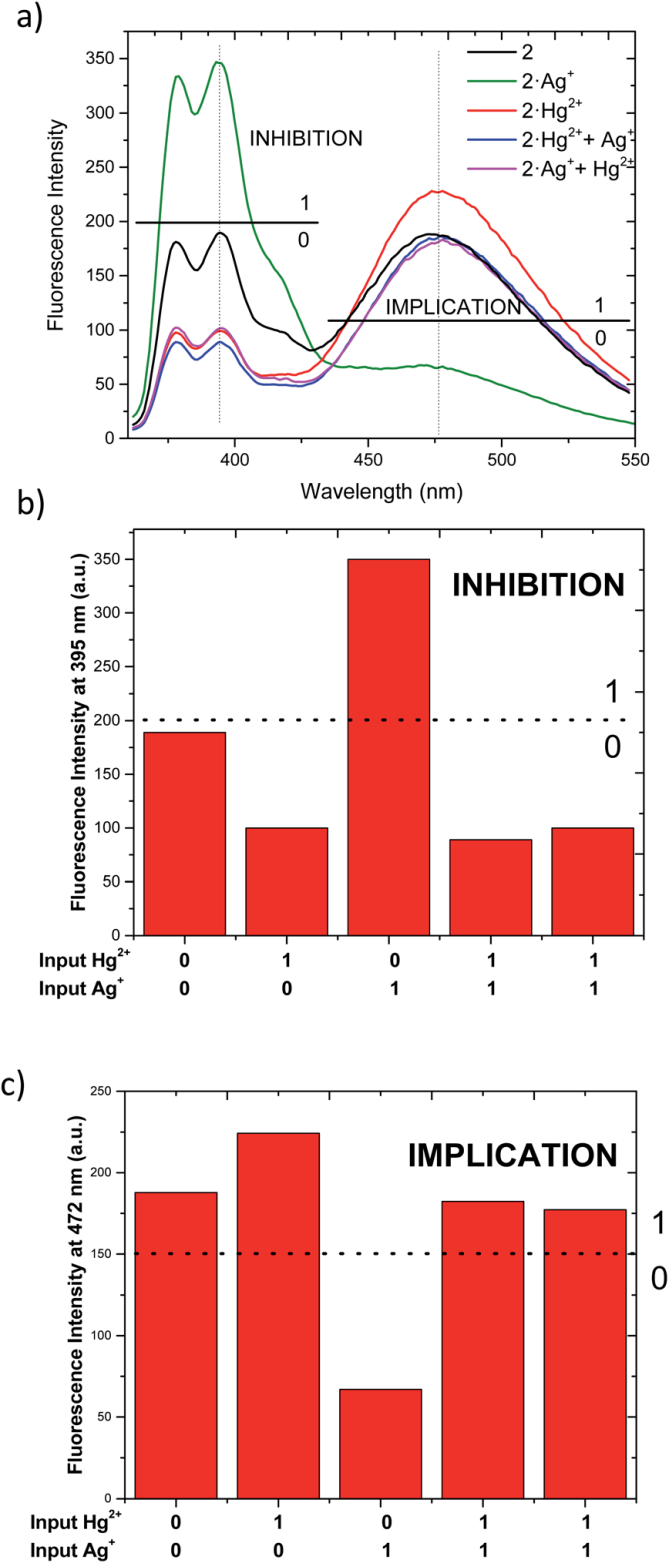
(a) Fluorescent emission changes of 2 at a concentration (1.15 μM) upon addition of Ag^+^ (1 eq.) and addition of Hg^2+^ (1 eq.) in CH_3_CN/DMSO (99 : 1). (b) Corresponding bar graph for INHIBITION. (c) Corresponding bar to IMPLICATION.

As described above, chemosensor 2 shows different response at pyrene monomer (*λ* = 395 nm) and excimer (*λ* = 472 nm) emission bands, when treated either with Ag^+^ or Hg^2+^. Therefore, we decide to evaluate its potential application as molecular logic gate at both emission wavelengths. Whether we situate the observation parameter for the output at one of monomer emission bands (*λ* = 395 nm), and define the threshold at fluorescence intensity 200 a.u., the initial output observed is “0” (Fig. 6a and b). Upon addition of 1 equivalent Ag^+^ (as input 1), the pyrene emission of 2 increases due to the quenching of excimer emission, resulting in “1” output. Upon addition of Hg^2+^ (input 2), the output becomes “0” due to the decrease of monomer emission. In the case where both inputs are added simultaneously (input 1 = input 2 = 1), the resulting emission output is again “0”, most likely due to chemosensor 2 stronger binding affinity for Hg^2+^. The resulting logic gate at this output corresponds to an INHIBIT-type, as summarized in Table 3 (see truth tables for all Boolean operations in Table S1†). When the observation parameter is situated at the excimer emission band (*λ* = 472 nm), and the threshold fluorescence intensity is defined at 150 a.u., the initial output observed is “1” (Fig. 6a and c), featuring one of the main prerequisites for IMPLICATION molecular logic gates. Addition of 1 equivalent Ag^+^ (input 1) results in quenching of excimer emission, being the observed output “0”. The output generated upon addition of 1 equivalent Hg^2+^ (input 2) is again “1” and similarly, the addition of both inputs simultaneously leads to an output “1”. Such combination allows for the configuration of an IMPLICATION-type logic gate (Tables 3 and S2†).

**Table 3:** Truth table for a two-input, one-output molecular switches

Input 1 (Ag^+^)^a^	Input 2 (Hg^2+^)^a^	Output (fluorescence at 395 nm)	Output (fluorescence at 472 nm)
0	0	0	1
1	0	1	0
0	1	0	1
1^b^	1^b^	0	1
		INHIBITION	IMPLICATION

a The input corresponds to 1 eq. of corresponding perchlorate salt in each case to a dissolution 1.15 μM.

b The addition was tested using alternating both input 1 and input 2.

## Conclusions

In the aim of developing excimer-based fluorescent chemosensors, we synthesized and characterised two pyrene-thiourea lower rim calix[4]arene derivatives. Only pyrene monomer emission was observed for 1, while 2 presented both monomer and excimer emission. This reveals the critical role that CH_2_ bridge plays for this type of excimer-based chemosensors. Fluorescent screening of 1 reveals a limited selectivity towards Hg^2+^, Cu^2+^ and Ag^+^. Same experiments for chemosensor 2 displayed ratiometric detection for Hg^2+^ and Ag^+^. Furthermore, this ratiometric variation is inverse for each cation, which allows for their selective detection. LODs for such complexes (2·Ag^+^ and 2·Hg^2+^) were both in the low nanomolar range (2.09 and 8.11 nM, respectively). Triggered by the complexity in identifying the coordination mode of 2, we performed computational conformational study of 2 and with the resulting complexes (2·Ag^+^ and 2·Hg^2+^). Boltzman distribution of 2 revealed that two conformations are present in the ground state. Study of these conformations with Ag^+^ and Hg^2+^ revealed that two differentiated binding sites are available in 2, where the stabilizing interactions were classified as *through-bond* or *through-space*, which have a different effect in the fluorescence of 2 and therefore, in the sensing mechanism. To the best of our knowledge, we have not found similar studies that address or propose these two binding sites for pyrene-calixarene derivatives, which might help further understanding of sensing mechanism involved in alike fluorescent chemosensors. Determination of *K*_app_ by Hill equation reveals that 2 interacts stronger with Hg^2+^ than with Ag^+^, which is in agreement with stability of 2·Ag^+^ and 2·Hg^2+^ complexes revealed by DFT calculations.

Additionally, chemosensor 2 was able to perform INHIBIT-type and IMPLICATION-type logic operations when analysing either monomer or excimer emission, being the later scarce in comparison with other configurable molecular logic gates. We additionally demonstrate that 2 logic gates are able to operate with equimolar inputs. Further work will be address on improving the solubility towards more polar organic solvents or aqueous media of 2 by additional functionalisation at calix[4]arenes upper or lower rim.

In summary, we think this work could contribute to further development and understanding of excimer-based chemosensors.

## Experimental

### Materials and methods

#### Reagents

All materials including solvents and perchlorate salts were purchased from commercial suppliers (Sigma Aldrich, Merck, Toronto Research Chemicals) and used without further purification. Chemical reactions were monitored by thin-layer chromatography (TLC) and were visualized using UV light. TLC was carried out on aluminum sheets coated with Silica Gel 60 F254 Merck (0.25 mm). The product purification was done using silica gel column chromatography (chromatogel SDS silica 60 AC.C 70–200 μM).

#### Characterisation

^1^H NMR spectra were measured on a Bruker Avance III HD (300 MHz) using tetramethylsilane (TMS, *δ* = 0.00 ppm) as internal standard. ^13^C NMR spectra were recorded at 75.5 MHz. Coupling constants were given in Hz. The following notations were used: br-broad, s-singlet, d-doublet, t-triplet, q-quartet, m-multiplet. Mass spectra LC/MSD-TOF were obtained in Universidad de Sevilla (Spain). UV absorption measurements were carried out on an Agilent Carey 60 spectrophotometer using standard 1.00 cm quartz cells. Fluorescence spectra were measured on Agilent Carey Eclipse UV spectra were carried out at 9.0 μM. Fluorescence spectra were measured by exciting the solutions of 1 and 2 (1.15 μM) at 340 nm with bandwidths for excitation and emission of 10 and 10 nm (for 1) and 5 and 10 (for 2), respectively, recording the emission spectra in the 360–550 range. The concentration of the cation was varied from 0 to 3 equivalents during the titration experiments.

Screening assays were performed by dissolving 1 and 2 in DMSO (stock solution 25 mM) and stock solutions 25 mM of cations (Na^+^, K^+^, Li^+^, Mg^2+^, Ca^2+^, Mn^2+^, Fe^2+^, Co^2+^, Ni^2+^, Cu^2+^, Zn^2+^, Pb^2+^, Cd^2+^, Hg^2+^ and Ag^+^) as perchlorate salts were prepared in CH_3_CN.

The stoichiometry of complexes (2·Ag^+^, 2·Hg^2+^) was attempted to be determined by continuous variation method, Job's plot^71,72,86^ Equimolar solutions of 2 and each cation were mixed to a standard volume (3 mL) varying the molar ratio but keeping the total concentration of the species constant. These studies were conducted by fluorescence spectroscopy with the same experimental conditions described above for chemosensor 2.

The LOD for 2 was calculated in acetonitrile as 3 times the standard deviation of the blank on the slope of the linearity in the calibration curve.

The titration data of compound 2 with Ag^+^ and Hg^2+^ were fitted to the Hill plot, where [M] stands for concentration:
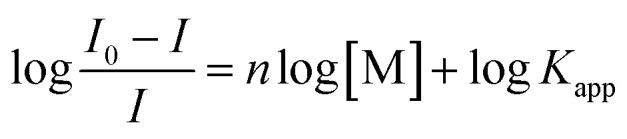


The Hill plot is the rearrangement of the Hill–Langmuir equation^75,76^ into a straight line. The slope of the line, named Hill coefficient (*n*) indicates the cooperativity of the binding process. From the plot is also possible to determine the apparent constant of complex equilibria.

### Quantum chemical calculations

#### Computational methods

The molecular geometry optimizations for gas phase 2 and the respective 2·Ag^+^ and 2·Hg^2+^ complexes in their ground states were carried out using the Grimme's dispersion-corrected (D3)^87^ hybrid B3LYP^88,89^ exchange-correlation functional (*i.e.* B3LYP-D3) combined with medium-sized basis set Def2SVP for both non-metal and metal atoms. In the case of metal centres was employed an effective core potential that describes the core electrons, replacing the 28 inner electrons for Ag^+^ and 60 for Hg^2+^ with a quasi-relativistic pseudopotential developed by Dolg and co-workers,^90^ while the valence electrons were quantum mechanically treated with the respective optimized valence basis set (7s6p5d1f)/[5s3p2d1f]-GTO for Ag^+^ and (7s6p5d1f)/[6s3p2d1f]-GTO for Hg^2+^ developed by Ahlrichs and co-workers.^91^ Moreover, harmonic frequency calculations were performed in order to confirm the optimized geometries as local minima on the potential energy surfaces. Using the ideal gas/rigid rotor/harmonic oscillator model, the frequency calculations also provide the thermal and entropic corrections for the free Gibbs energies at room temperature. The vertical excitation energies for singlet-excited states in solution were investigated within the TD-DFT coupled to implicit solvation model framework. To carry out this task the dispersion-corrected range-separated hybrid CAM-B3LYP^92^ exchange-correlation functional (*i.e.* CAM-B3LYP-D3) together with Def2SVP basis set was employed, while the solvation model density (SMD)^93^ continuum solvation model using acetonitrile as solvent was applied to describe solute–solvent interactions. All calculations presented here were performed with the Gaussian09 suite of programs.^91^ CYLview was employed to depict molecular structures.^92^

### Synthesis

#### Calix[4]arene derivative 1

1-Pyreneisothiocyanate (0.115 g, 0.53 mmol) and triethylamine (20 μL) were added to a solution of calixarene (0.20 g, 0.244 mmol) in dry CH_2_Cl_2_ (10 mL). The reaction mixture was stirred by 48 h at room temperature. A white precipitate was observed upon completion of the reaction. The precipitate was filtered and washed with cold CH_2_Cl_2_ to get a white amorfous solid product. 0.11 g (32.8%).

^1^H-NMR (300 MHz, CDCl_3_-DMSO-*d*^6^, 298 K): *δ* (ppm): 10.44 (s, 2H, NHAr), 8.10 (bs, 4H, Ar–pyrene), 8.23 (m, 4H, Ar–pyrene), 8.05 (t, 2H, ^3^*J*_H,H_ = 7.50 Hz, Ar–pyrene), 7.97 (d, 2H, ^3^*J*_H,H_ = 8.0 Hz, Ar–pyrene), 7.79 (d, 2H, ^3^*J*_H,H_ = 8.0 Hz, Ar–pyrene), 7.67 (m, 4H, Ar–pyrene), 7.48 (bs, 2H, CH_2_NHAr), 7.00 (s, 2H, OH), 6.63 (s, 4H, ArCalix), 6.44 (s, 4H, ArCalix), 3.73 (bs, 4H, CH_2_O), 3.30–3.18 (m, 8H, ArCH_2_Ar, CH_2_NH), 2.41 (bd, 2H, ^2^*J*_H,H_ = 13.0 Hz, ArCH_2_Ar), 1.11 (s, 18H, (CH_3_)_3_), 0.96 (s, 18H, (CH_3_)_3_).

^13^C-NMR: (75.5 MHz, CDCl_3_-DMSO-*d*^6^): *δ* (ppm): 182.1 (CS), 148.1, 148.0, 147.9, 141.9, 133.3, 131.0, 130.8, 130.1, 128.4, 127.5, 127.1, 129.9, 126.6, 125.8, 125.5, 125.4, 124.9, 124.8, 124.6, 124.0, 122.0 (Ar), 74.6 (OCH_2_), 54.6 (CH_2_NH), 34.3 (C(CH_3_)_3_), 33.7 (ArCH_2_Ar), 31.6, 31.0 (C(CH_3_)_3_). HRMS LC/MSD-TOF: *m*/*z* calcd for C_82_H_84_N_4_NaO_4_S_2_ 1275.5832. Found: 1275.5826.

#### Calix[4]arene derivative 2

1-Pyrenemethyl isothiocyanate (90 mg, 0.34 mmol) and triethylamine (16 μL) were added to a solution of calixarene (0.12 g, 0.16 mmol) in CH_2_Cl_2_ (10 mL). The reaction mixture was stirred by 48 h at room temperature. A white precipitate was observed upon completion of the reaction. The precipitate was filtered off and washed with cold CH_2_Cl_2_ to get a white solid product. 80 mg (39%).

^1^H-NMR (300 MHz, CDCl_3-_DMSO-*d*^6^, 298 K): *δ* (ppm): 8.27–7.70 (m, 20H, Ar–pyrene, CSNH), 6.74 (s, 4H, s, 4H, ArCalix), 6.60 (s, 4H, ArCalix), 6.42 (s, 2H, OH), 5.37 (s, 4H, ArC*H*_2_NH), 3.66 (bs, 4H, CH_2_O), 3.33 (m, 8H, ArCH_2_Ar, OCH_2_C*H*_2_NH), 2.74 (d, 4H, ^2^*J*_H,H_ = 13.4 Hz, ArCH_2_Ar), 1.19 (s, 18H, (CH_3_)_3_), 0.94 (s, 18H, (CH_3_)_3_). ^13^C-NMR (75.5 MHz, CDCl_3-_DMSO-*d*^6^, 298 K): *δ* (ppm): 182.7 (CS), 148.1, 148.0, 147.6, 143.3, 132.7, 131.1, 130.9, 130.6, 128.7, 128.3, 127.5, 127.4, 127.3, 126.1, 125.8, 125.5, 125.4, 125.3, 124.9, 124.8, 124.7, 122.6 (Ar), 74.67 (OCH_2_), 46.7 (ArC*H*_2_NH), 44.0 (OCH_2_*C*H_2_NH), 34.0, 33.8 (*C*(CH_3_)_3_), 31.5 (ArCH_2_Ar), 31.5, 30.9 (C(CH_3_)_3_). HRMS LC/MSD-TOF: *m*/*z* calcd for C_84_H_88_N_4_NaO_4_S_2_ 1303.6145. Found: 1303.6123.

## Conflicts of interest

There are no conflicts to declare.

## Supplementary Material

RA-010-D0RA04092D-s001

## References

[cit1] Valeur B., Leray I. (2000). Coord. Chem. Rev..

[cit2] Butt S. B., Riaz M. (2009). J. Liq. Chromatogr. Relat. Technol..

[cit3] Wang L., Peng X., Fu H., Huang C., Li Y., Liu Z. (2020). Biosens. Bioelectron..

[cit4] Järup L. (2003). Br. Med. Bull..

[cit5] Kim J. S., Quang D. T. (2007). Chem. Rev..

[cit6] Mako T. L., Racicot J. M., Levine M. (2019). Chem. Rev..

[cit7] De Acha N., Elosúa C., Corres J. M., Arregui F. J. (2019). Sensors.

[cit8] Barillo D. J., Marx D. E. (2014). Burns.

[cit9] Dargo H., Ayaliew A., Kassa H. (2017). Sustain. Mater. Technol..

[cit10] Purcell T. W., Peters J. J. (1998). Environ. Toxicol. Chem..

[cit11] Lansdown A. B. G. (2007). Crit. Rev. Toxicol..

[cit12] Drake P. L., Hazelwood K. J. (2005). Ann. Occup. Hyg..

[cit13] Bernhoft R. A. (2012). J. Environ. Public Health.

[cit14] Langford N. J., Ferner R. E. (1999). J. Hum. Hypertens..

[cit15] Cappelletti S., Piacentino D., Fineschi V., Frati P., Errico D., Aromatario M., Cappelletti S., Piacentino D., Fineschi V., Frati P. (2019). Crit. Rev. Toxicol..

[cit16] El-safty S. A., Shenashen M. A. (2012). Trends Anal. Chem..

[cit17] Li Y., Yuan J., Xu Z. (2019). J. Anal. Methods Chem..

[cit18] Lin W. C., Wu C. Y., Liu Z. H., Lin C. Y., Yen Y. P. (2010). Talanta.

[cit19] Wang G. K., Mi Q. L., Zhao L. Y., Hu J. J., Guo L. E., Zou X. J., Liu B., Xie X. G., Zhang J. F., Zhao Q. H., Zhou Y. (2014). Chem. –Asian J..

[cit20] Velmurugan K., Raman A., Easwaramoorthi S., Nandhakumar R. (2014). RSC Adv..

[cit21] Hao J. N., Yan B. (2015). J. Mater. Chem. A.

[cit22] Joseph R., Ramanujam B., Acharya A., Rao C. P. (2009). J. Org. Chem..

[cit23] Liu L., Zhang D., Zhang G., Xiang J., Zhu D. (2008). Org. Lett..

[cit24] Choi S., Lee G., Park I. S., Son M., Kim W., Lee H., Lee S. Y., Na S., Yoon D. S., Bashir R., Park J., Lee S. W. (2016). Anal. Chem..

[cit25] Lim Z., Smith D. G., Kolanowski J.
L., Mattison R. L., Knowles J. C., Baek S. Y., Chrzanowski W., New E. J. (2018). J. R. Soc. Interface.

[cit26] Kumar M., Kumar R., Bhalla V. (2013). Tetrahedron Lett..

[cit27] Yang M. H., Thirupathi P., Lee K. H. (2011). Org. Lett..

[cit28] Valeur B. and Berberan-Santos M. N., Molecular Fluorescence, 2012

[cit29] Ma J., Song M., Boussouar I., Tian D., Li H. (2015). Supramol. Chem..

[cit30] Wu D., Sedgwick A. C., Gunnlaugsson T., Akkaya E. U., Yoon J., James T. D. (2017). Chem. Soc. Rev..

[cit31] Song M., Sun Z., Han C., Tian D., Li H., Kim J. S. (2014). Chem. –Asian J..

[cit32] Ludwig R., Dzung N. T. K. (2002). Sensors.

[cit33] Mohapatra R. K., Das P. K., Pradhan M. K., El-Ajaily M. M., Das D., Salem H. F., Mahanta U., Badhei G., Parhi P. K., Maihub A. A., E-Zahan M. K. (2019). Comments Inorg. Chem..

[cit34] Jose D. A., Kumar D. K., Ganguly B., Das A. (2005). Tetrahedron Lett..

[cit35] Carbajo-Gordillo A. I., Rodríguez-Lavado J., Jiménez Blanco J. L., Benito J. M., Di Giorgio C., Vélaz I., De Ilarduya C. T., Ortiz Mellet C., García Fernández J. M. (2019). Chem. Commun..

[cit36] Díaz-Moscoso A., Le Gourriérec L., Gómez-García M., Benito J. M., Balbuena P., Ortega-Caballero F., Guilloteau N., Di Giorgio C., Vierling P., Defaye J., Mellet C. O., Fernández J. M. G. (2009). Chem. –Eur. J..

[cit37] Díaz-Moscoso A., Balbuena P., Gómez-García M., Ortiz Mellet C., Benito J. M., Le Gourriérec L., Di Giorgio C., Vierling P., Mazzaglia A., Micali N., Defaye J., García Fernández J. M. (2008). Chem. Commun..

[cit38] Nimse S. B., Kim T. (2013). Chem. Soc. Rev..

[cit39] Zhang L., Jian Y., Wang J., He C., Li X., Liu T., Duan C. (2012). Dalt. Trans..

[cit40] Vonlanthen M., Connelly C. M., Deiters A., Linden A., Finney N. S. (2014). J. Org. Chem..

[cit41] Siu H., Duhamel J. (2004). Macromolecules.

[cit42] Winnik F. M. (1993). Chem. Rev..

[cit43] Pizarro J., Flores E., Jimenez V., Maldonado T., Saitz C., Vega A., Godoy F., Segura R. (2019). Sens. Actuators, B.

[cit44] Gómez-Machuca H., Quiroga-Campano C., Jullian C., De La Fuente J., Pessoa-Mahana H., Escobar C. A., Dobado J. A., Saitz C. (2015). J. Incl. Phenom. Macrocycl. Chem..

[cit45] Gruber T., Seichter W., Weber E. (2008). Supramol. Chem..

[cit46] Kołaski M., Arunkumar C. R., Kim K. S. (2013). J. Chem. Theory Comput..

[cit47] Bandela A. K., Bandaru S., Rao C. P. (2015). Chem. –Eur. J..

[cit48] Bandela A., Chinta J. P., Rao C. P. (2011). Dalt. Trans..

[cit49] Lohar L., Safin D. A., Sengupta A., Chattopadhyay A., Matalabos J. S., Babashkina M. G., Robeyns K., Mitoraj M. P., Kubisiak P., Garcia Y., Das D. (201). Chem. Commun.

[cit50] Gunnlaugsson T., James T. D., Yoon J., Akkaya E. U., Akkaya E. U. (2017). Chem. Soc. Rev..

[cit51] Quiroga-Campano C., Gómez-Machuca H., Moris S., Jara P., De la Fuente J. R., Pessoa-Mahana H., Jullian C., Saitz C. (2017). J. Mol. Struct..

[cit52] Frazier K. M., Swager T. M. (2013). Anal. Chem..

[cit53] Wang G. K., Mi L., Zhao Y., Hu J., Guo L. E., Zou J. (2014). Chem.–Asian J..

[cit54] Sahin O., Yilmaz M. (2011). Tetrahedron.

[cit55] Kumar M., Kumar R., Bhalla V. (2013). Tetrahedron Lett..

[cit56] Nishizawa S., Teramae N. (1997). Anal. Sci..

[cit57] Kumar R., Bhalla V., Kumar M. (2008). Tetrahedron.

[cit58] Nohta H., Satozono H., Koiso K., Yoshida H., Ishida J., Yamaguchi M. (2000). Anal. Chem..

[cit59] Jiang Y. L., Broome A.-M. (2019). ACS Sensors.

[cit60] Zhao M., Zhou X., Tang J., Deng Z., Xu X., Chen Z., Li X., Yang L., Ma L. J. (2017). Spectrochim. Acta, Part A.

[cit61] Talanova G. G., Elkarim N. S. A., Talanov V. S., Bartsch R. A. (1999). Anal. Chem..

[cit62] Ben Othman A., Jeong W. L., Wu J. S., Jong S. K., Abidi R., Thuéry P., Strub J. M., Van Dorsselaer A., Vicens J. (2007). J. Org. Chem..

[cit63] Kumar R., Sharma A., Singh H., Suating P., Kim H. S., Sunwoo K., Shim I., Gibb B. C., Kim J. S. (2019). Chem. Rev..

[cit64] Bai C. B., Xu P., Zhang J., Qiao R., Chen M. Y., Mei M. Y., Wei B., Wang C., Zhang L., Chen S. S. (2019). ACS Omega.

[cit65] Kumar M., Kumar N., Bhalla V., Kaur A. (2013). Supramol. Chem..

[cit66] Hsieh Y. C., Chir J. L., Yang S. T., Chen S. J., Hu C. H., Wu A. T. (2011). Carbohydr. Res..

[cit67] Dhir A., Bhalla V., Kumar M. (2008). Org. Lett..

[cit68] Khantaw T., Boonmee C., Tuntulani T., Ngeontae W. (2013). Talanta.

[cit69] Zhu M., Zhou Y., Yang L., Li L., Qi D., Bai M., Chen Y., Du H., Bian Y. (2014). Inorg. Chem..

[cit70] Thordarson P. (2011). Chem. Soc. Rev..

[cit71] Brynn Hibbert D., Thordarson P. (2016). Chem. Commun..

[cit72] Ulatowski F., Dabrowa K., Bałakier T., Jurczak J. (2016). J. Org. Chem..

[cit73] Tong D., Duan H., Zhuang H., Cao J., Wei Z., Lin Y. (2014). RSC Adv..

[cit74] De Juan A., López-Moreno A., Calbo J., Ortí E., Pérez E. M. (2015). Chem. Sci..

[cit75] Gesztelyi R., Zsuga J., Kemeny-Beke A., Varga B., Juhasz B., Tosaki A. (2012). Arch. Hist. Exact Sci..

[cit76] Ercolani G. (2003). J. Am. Chem. Soc..

[cit77] Huenerbein R., Grimme S. (2008). Chem. Phys..

[cit78] Azumi T., Armstrong A. T., Mcglynn S. P. (1964). J. Chem. Phys..

[cit79] Birks J. B., Dyson D. J., Munro I. H. (1963). Proc. R. Soc. London, Ser. A.

[cit80] Unger-Angel L., Motiei L., Margulies D. (2019). Front. Chem..

[cit81] Akkaya E. U., Katz E., Pischel U. (2017). ChemPhysChem.

[cit82] Tzeli D., Petsalakis I. D. (2019). J. Chem..

[cit83] Rurack K., Trieflinger C., Koval’chuck A., Daub J. (2007). Chem.–Eur. J..

[cit84] Deng H.-H., Wu G.-W., Lin X.-Q., Xu X.-W., Liu A.-L., Xia X.-H., Chen W. (2015). RSC Adv..

[cit85] Gotor R., Costero A. M., Gil S., Parra M., Gaviña P., Rurack K. (2013). Chem. Commun..

[cit86] Renny J. S., Tomasevich L. L., Tallmadge E. H., Collum D. B. (2013). Angew. Chem., Int. Ed. Engl..

[cit87] Grimme S., Antony J., Ehrlich S., Krieg H. (2010). J. Chem. Phys..

[cit88] Becke A. D. (1993). J. Chem. Phys..

[cit89] Lee C., Hill C., Carolina N. (1997). Phys. Rev..

[cit90] Andrae D., Häuβermann U., Dolg M., Stoll H., Preub H. (1990). Theor. Chim. Acta.

[cit91] Eichkorn K., Weigend F., Treutler O., Ahlrichs R. (1997). Theor. Chem. Acc..

[cit92] Yanai T., Tew D. P., Handy N. C. (2008). Chem. Phys. Lett..

[cit93] Marenich A. V., Cramer C. J., Truhlar D. G. (2009). J. Phys. Chem. B.

