# Supplementary material for: Elucidating sensing mechanisms of a pyrene excimer-based calix[4]arene for ratiometric detection of Hg(ii) and Ag(i) and chemosensor behaviour as INHIBITION or IMPLICATION logic gates
Source: RSC Adv. 2020 Jun 8;10(37):21963–73. doi: 10.1039/d0ra04092d (PMC9054513; doi:10.1039/d0ra04092d)
Supplement: RA-010-D0RA04092D-s001 [file RA-010-D0RA04092D-s001.pdf]

Electronic Supplementary Material (ESI) for RSC Advances.
This journal is © The Royal Society of Chemistry 2020

Supporting Information

Elucidating sensing mechanisms of a pyrene-excimer based calix[4]arene for ratiometric detection of Hg(II) and Ag (I) and chemosensor behaviour as INHIBITION or IMPLICATION logic gates

Julio Rodríguez-Lavado*[a], Alejandro Lorente[a], Erick Flores[b], Andrés Ochoa[a], Fernando Godoy[b], Pablo Jaque[a], Claudio Saitz*[a]

[a] Departamento de Química Orgánica y Fisicoquímica, Facultad de Ciencias Químicas y Farmacéuticas, Universidad de Chile, Olivos 1007, Santiago, Chile
[b]Departamento de Química de los Materiales, Universidad de Santiago de Chile, Libertador Bernardo O´Higgins 3363, Santiago, RM

email of the corresponding authors: julio.rodriguez@ciq.uchile.cl, clsaitz@ciq.uchile.cl

I. Experimental Information

a) **Figures S1 to S7 and Table S1……………………….…..…2-5**

b) **NMR Spectra…………………………………………….....6-9**

c) **Mass Spectra…………………………………………………0**

II. Conformational Analysis of derivative 1 and Frontier Molecular Orbitals

Conformers derivative 1, Figure S13…………………………10

Frontier Molecular Orbitals of 2a, Figure S14 ………………11

III. Boolean Operations for two inputs…………………………...12

I. EXPERIMENTAL INFORMATION

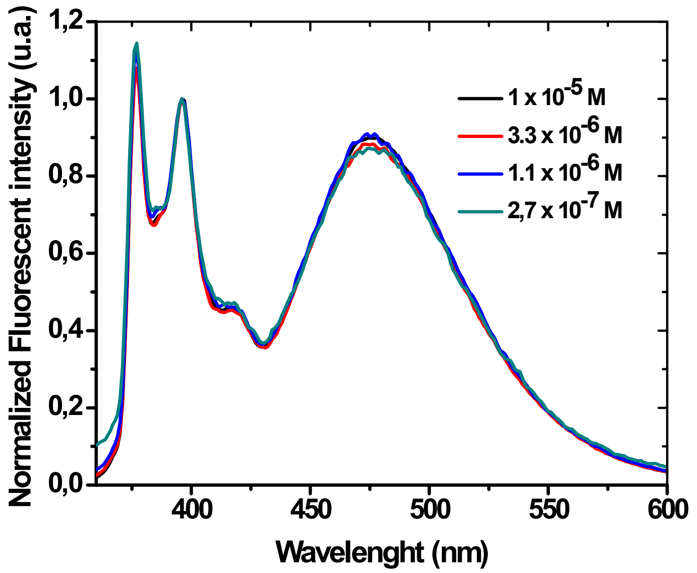

Figure S1. Normalized fluorescence intensities of **2** at different dilutions (CH_3CN/DMSO 99:1).

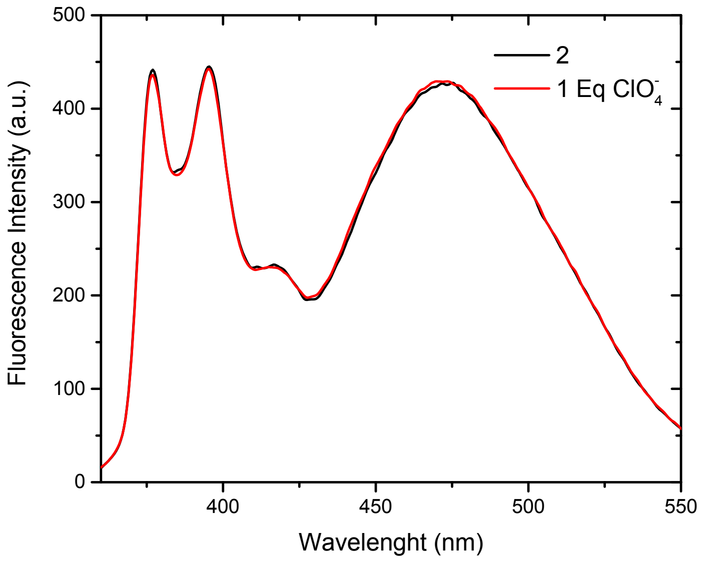

Figure S2. Fluorescence spectra of **2** (1.15 μM in CH_3C/DMSO 99:1) in presence of 2 equilvalents of NBu_4ClO_4.

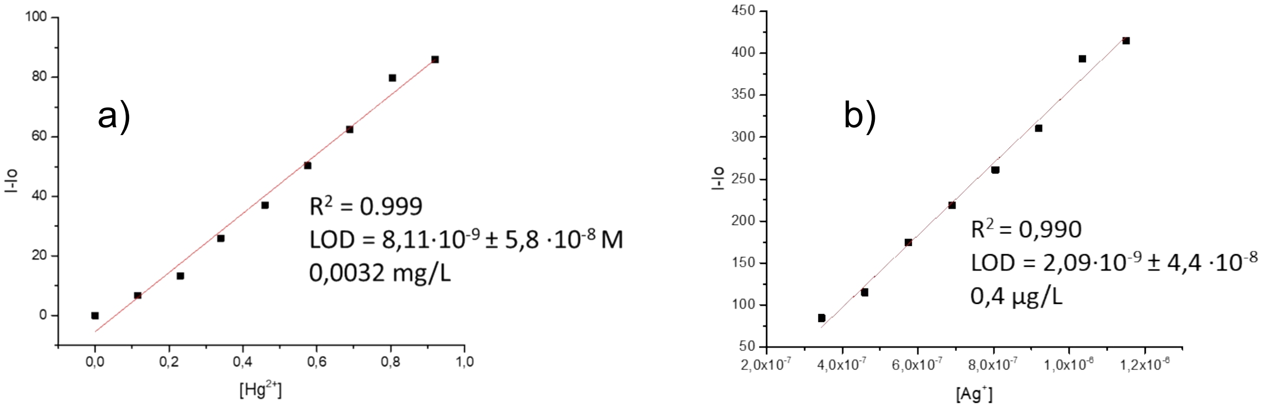

Figure S3. Calibration curve of **2** with Hg^{2+} (a) and Ag+ (b) in (CH_3CN/DMSO 99:1) for LOD determination.

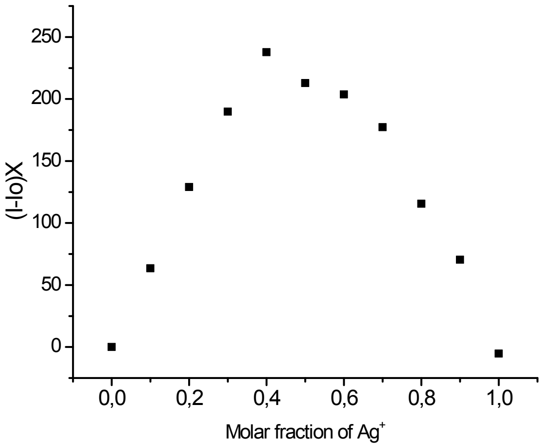

Figure S4. Job Plot for a complex of compound **2** and Ag^+ ion (λ=372 nm, CH_3CN/DMSO 99:1)

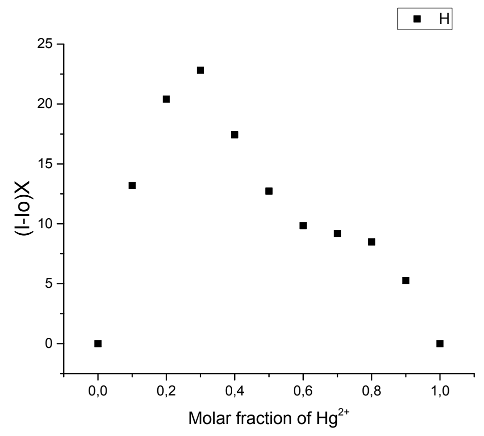

Figure S5. Job Plot for a complex of compound **2** and Hg^{2+} ion (λ=372 nm, CH_3CN/DMSO 99:1)

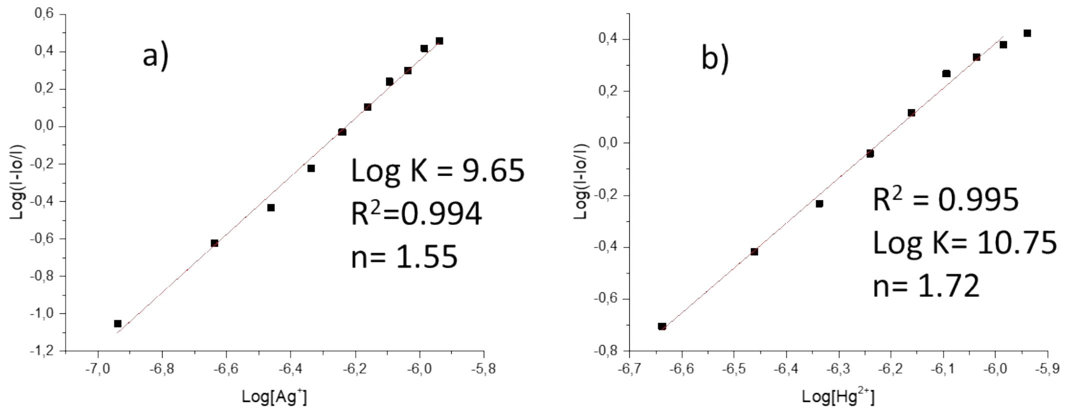

Figure S6. Hill Plots for the complexes 2:Ag^+ (a) and 2:Hg^{2+} (b) in CH_3CN/DMSO (99:1)

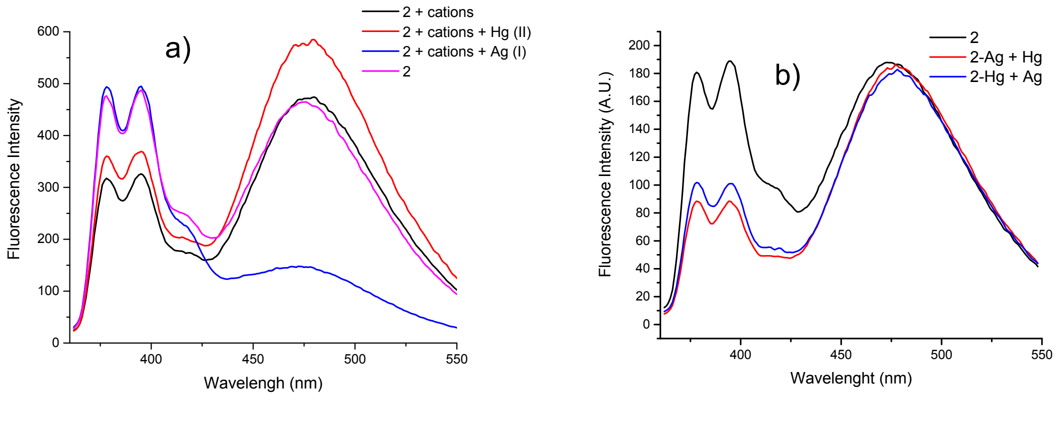

Figure S7. a) Interference studies of **2** in presence of Ag^+, Hg^{2+}, and cation mixture and (b) competitive interferent study of **2** with Ag^+ and Hg^{2+}

Ref	LOD Hg^{2+} (nM)	LOD Ag^{+} (nM)	Solvent
65	70	-	THF
67	100	-	THF/H_2O 9:1
64	36	-	HEPES/CH_3CN 3:7
66	12500	-	MeOH
69	-	68	Acetic/Acetate Buffer
68	-	200	DMF
**2**[a]	2.09	8.11	CH_3CN

Table S1. Comparative Results for Hg^{2+} and Ag^{+} detection probes. [a]Compound **2** studied in the present work

a) ^{1}H and ^{13}C Spectra

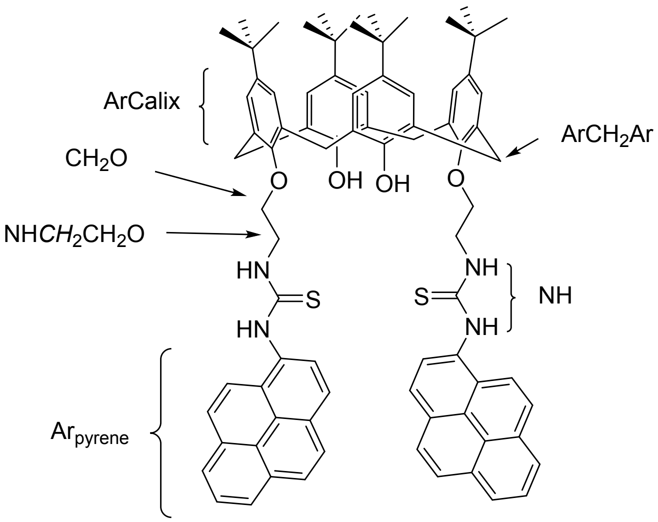

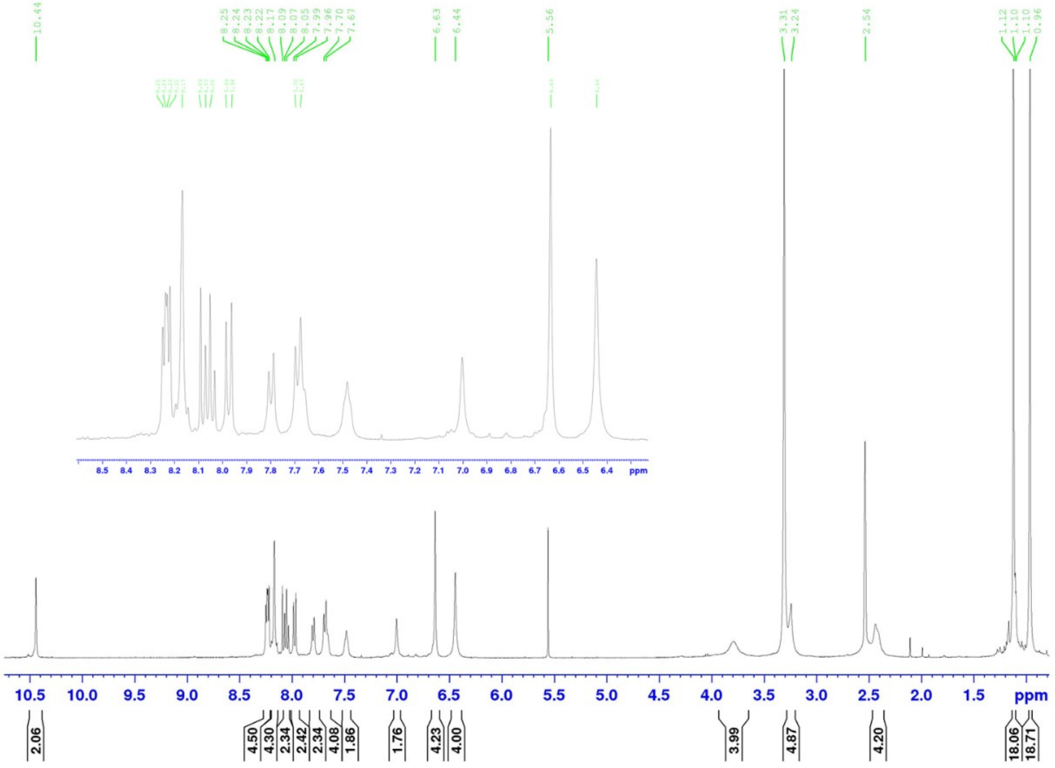

Figure S8. ^{1}H NMR Spectrum (300 MHz, 298 K) of compound **1**.

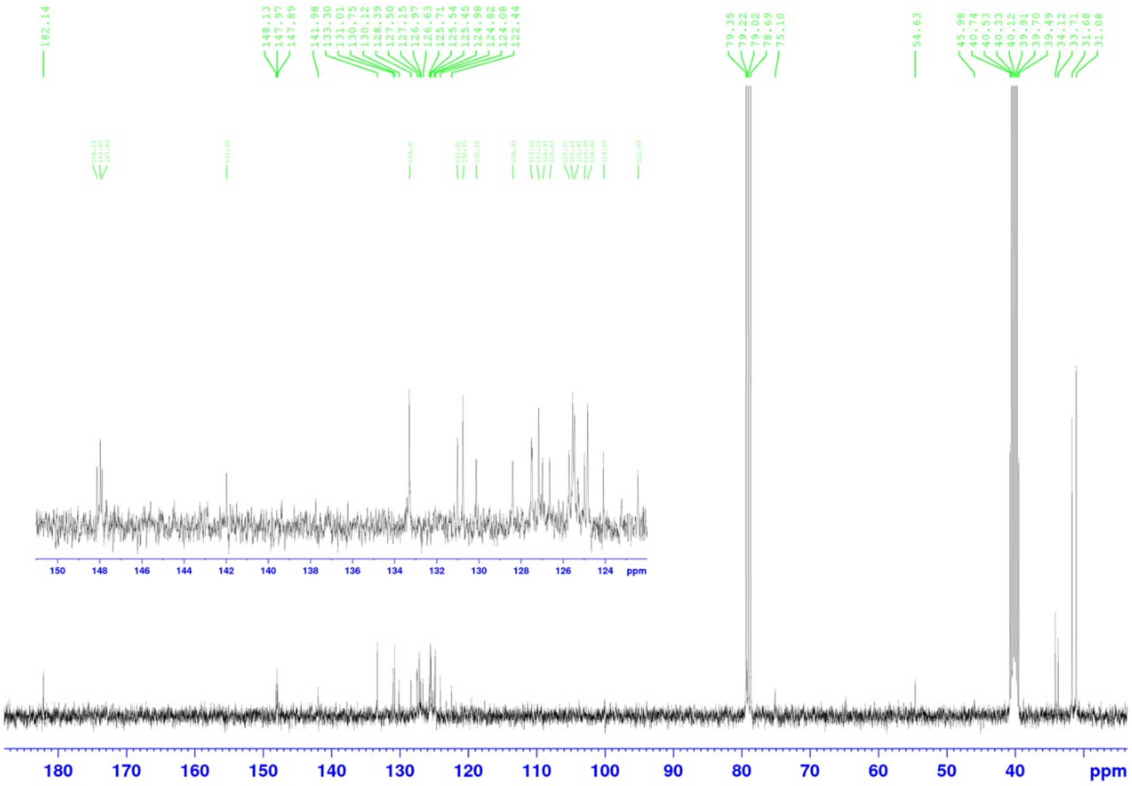

Figure S9. ^{3}C NMR Spectrum (75.5 MHz, 298 K) of compound **1**.

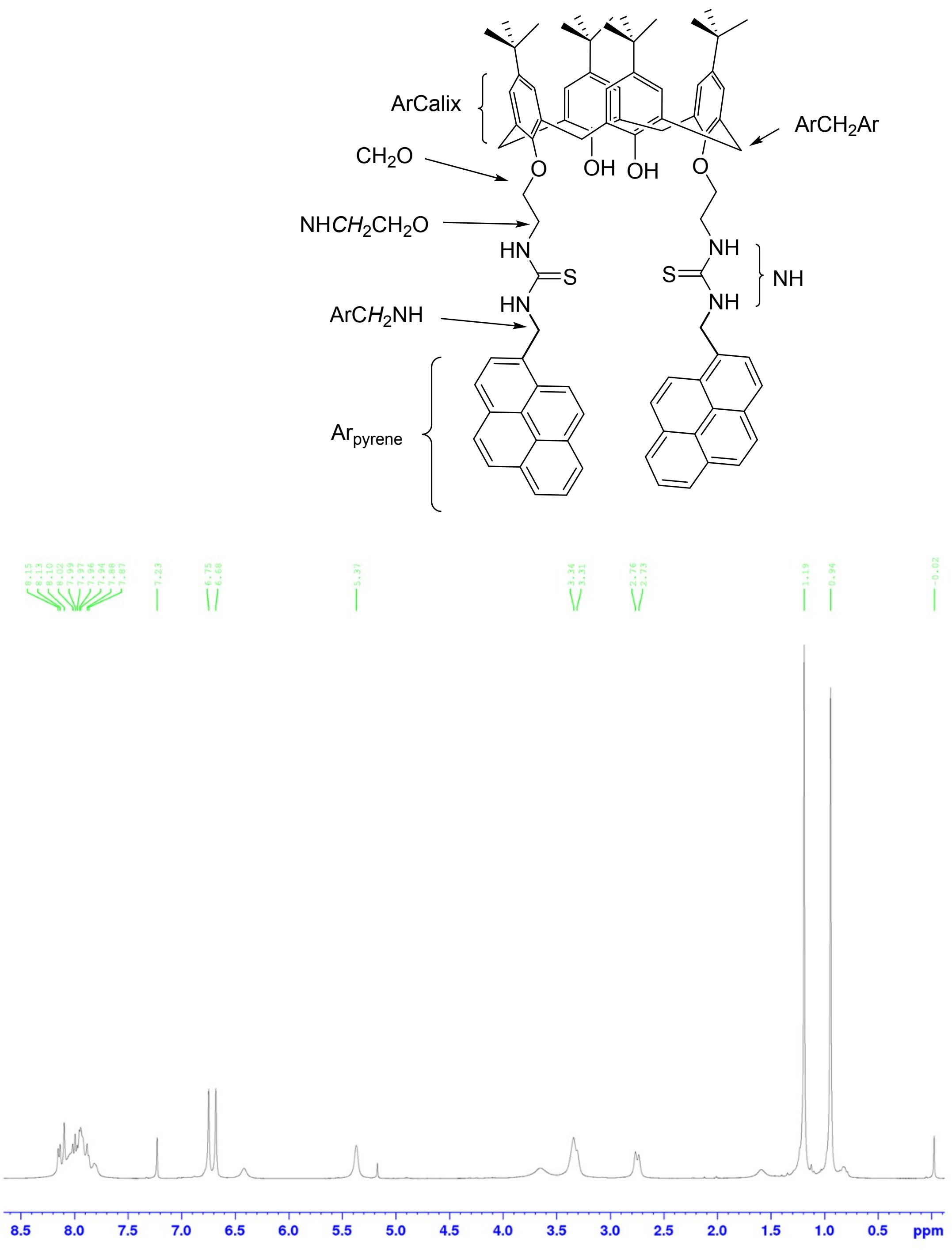

Figure S10. ^{1}H NMR Spectrum (300 MHz, 298 K) of compound **2**.

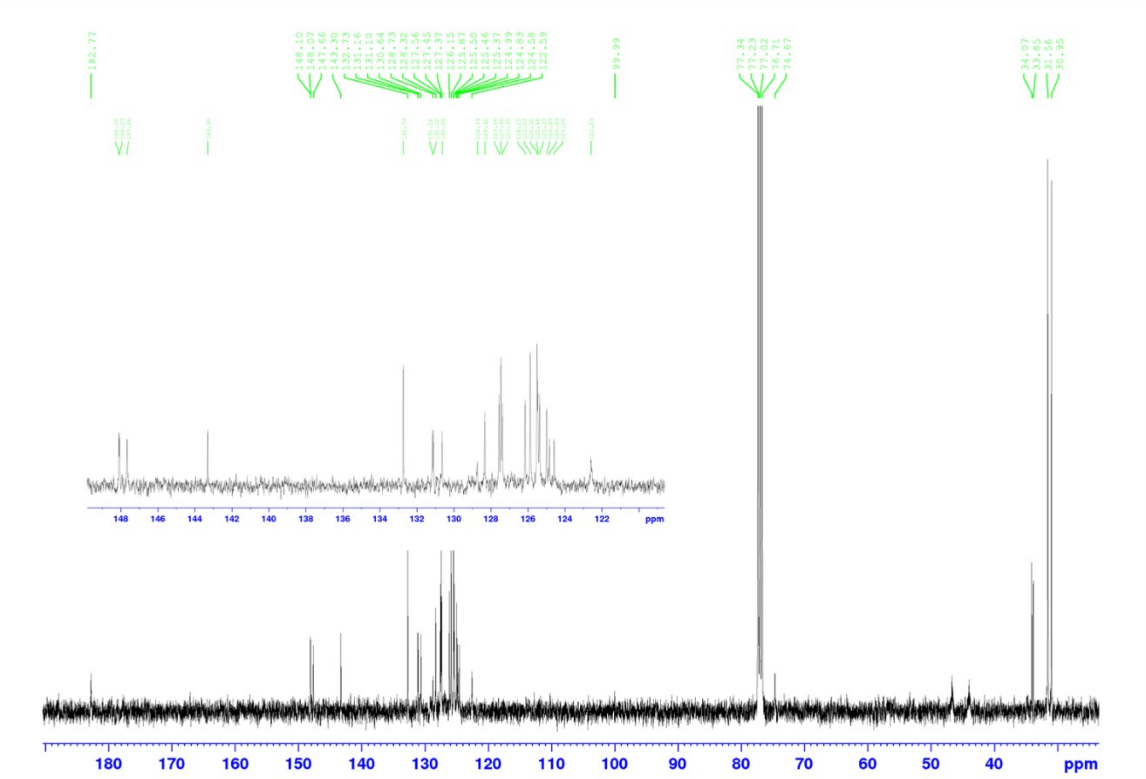

Figure S11. ^{13}C NMR Spectrum (75.5 MHz, 298 K) of compound **2**.

b) Mass Spectra

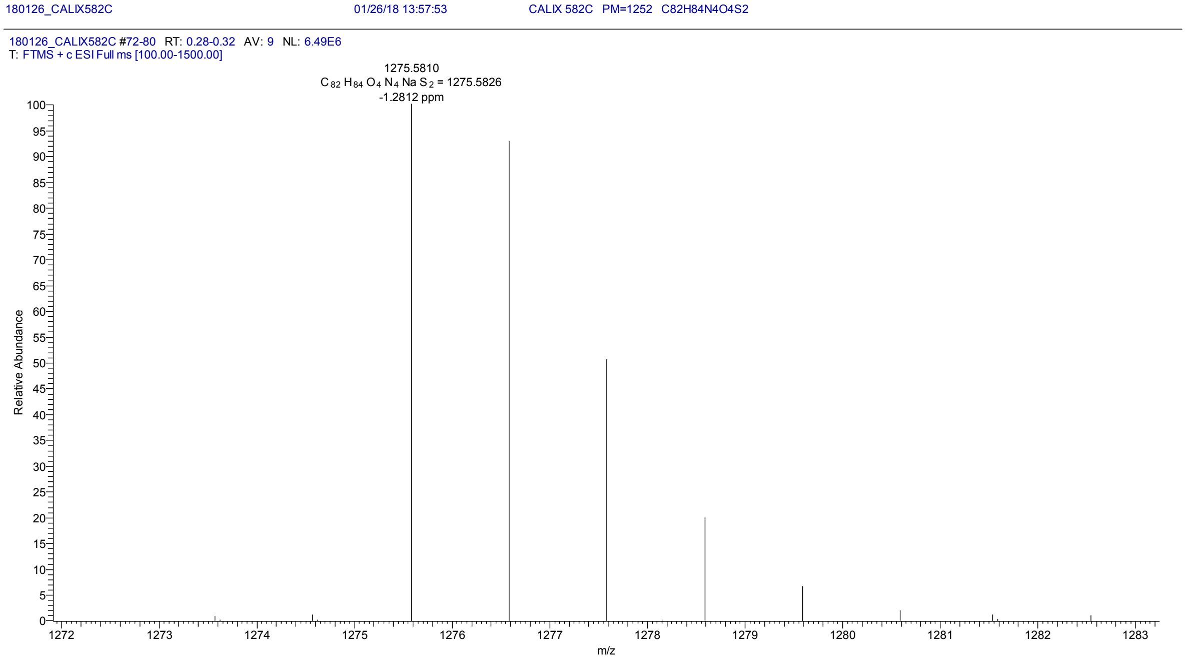

Figure S12. HR2MS (ESI) of compound **1**

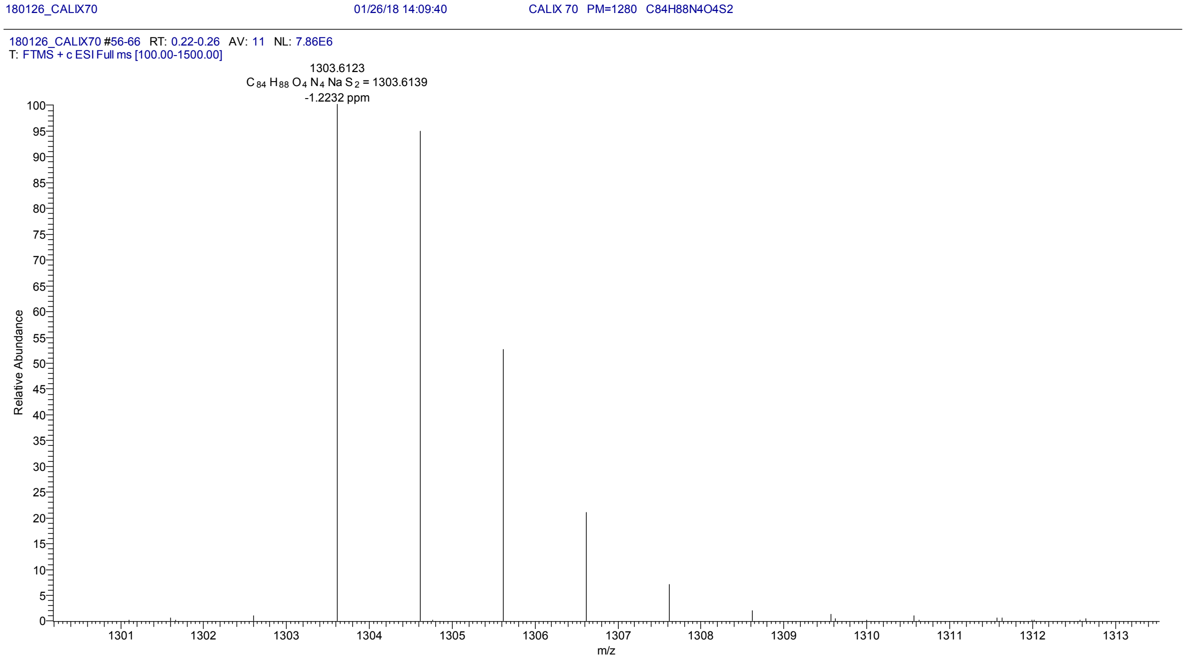

Figure S13. HR-MS (ESI) of compound **2.**

II. Conformational analysis of derivative 1 and Frontier Molecular Orbitals

1a **1b** **1c**

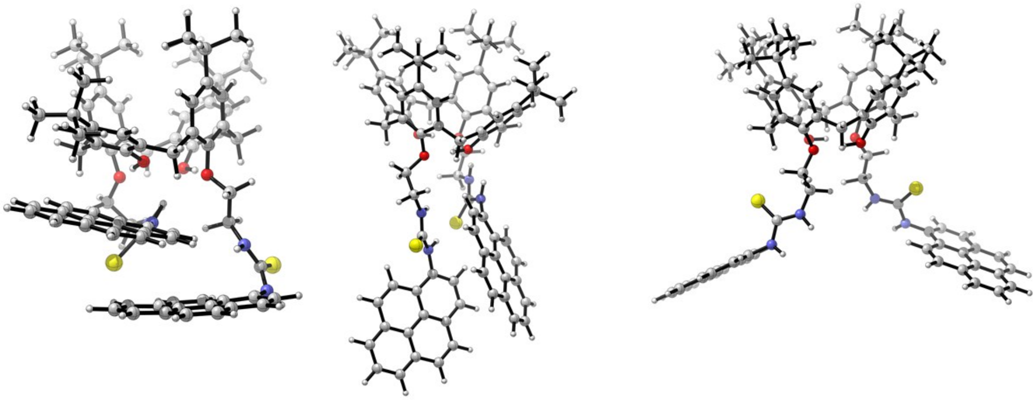

	1a	1b	1c
ΔG	0.0	4.09	10.91
(% pop)	100	0	0

Figure S14. Highest (H-1 and H) and lowest (L and L+1) molecular orbitals of **2a**.

Frontiers Molecular Orbitals of 2a

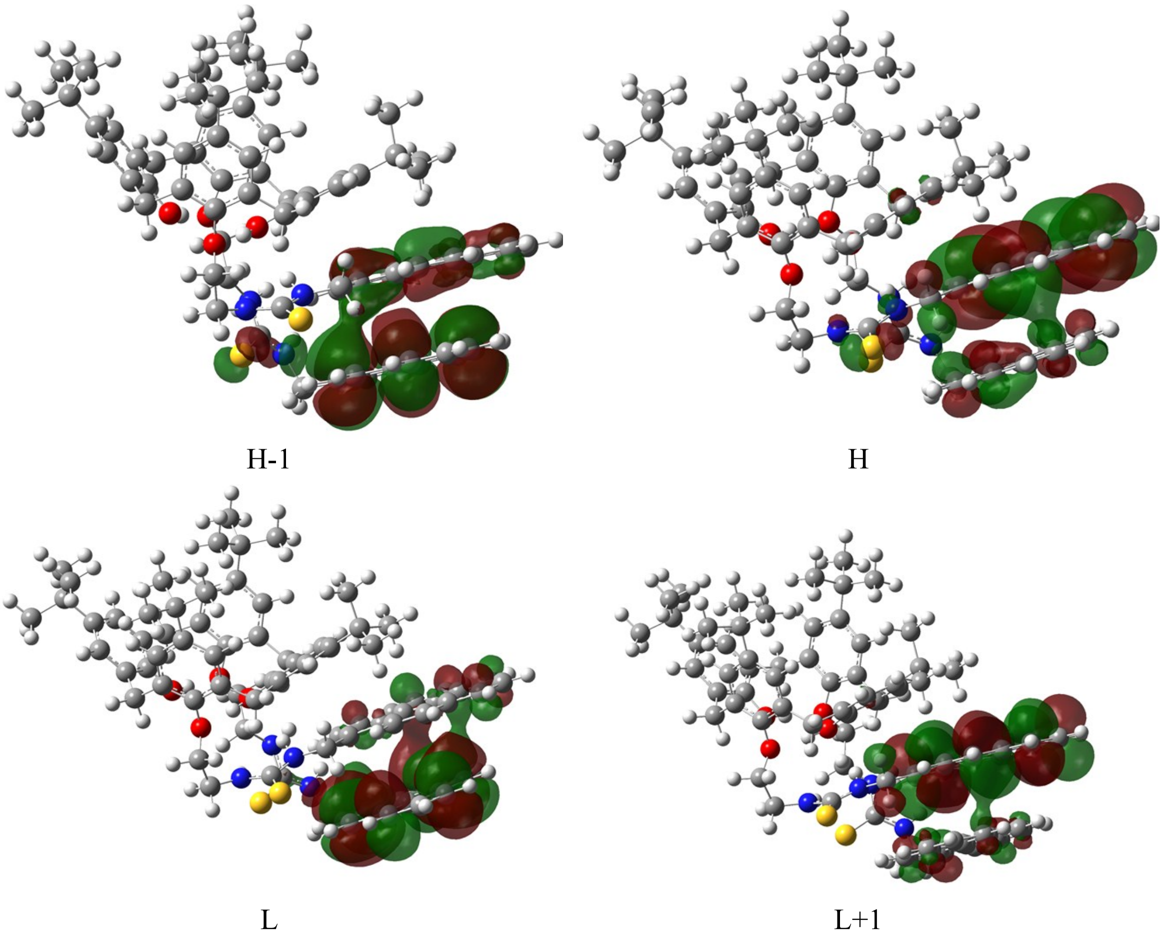

Figure S15. Highest (H-1 and H) and lowest (L and L+1) molecular orbitals of **2a**.

III. Boolean operations for two inputs

Table S2. Table of Boolean Operations for two inputs [1]

Operation N°	Commutative to operation N°	Result for				Entry	Meaning	Logic gate
		A=0 B=0	A=0 B=1	A=1 B=0	A=1 B=1			
0	15	0	0	0	0	’0’ constant	‘0’ constant	ZERO
1	14	0	0	0	1	Conjuction	A and B	AND
2	-	0	0	1	0	Exclusion	A excludes B	INHIBIT
3	12	0	0	1	1	1st variable	A	ID A
4	-	0	1	0	0	Exclusion	B excluding A	INHIBIT
5	10	0	1	0	1	2nd variable	B	ID B
6	9	0	1	1	0	Nonequivalence	Either A or B	XOR
7	8	0	1	1	1	Disjunction	A or B	OR
8	7	1	0	0	0	Nondisjunction	Neither A or B	NOR
9	6	1	0	0	1	Equivalence operation	A equivalent to B	EQU
10	5	1	0	1	0	Negation of variable B	Not B	INV B
11	-	1	0	1	1	Implication	B implies A	IMPLICATION
12	3	1	1	0	0	Negation of variable A	Not A	INV A
13	-	1	1	0	1	Implication	A implies B	IMPLICATION
14	1	1	1	1	0	Nonconjunction	No both A and B	NAND
15	0	1	1	1	1	‘1’ constant	‘1’ constant	ONE
